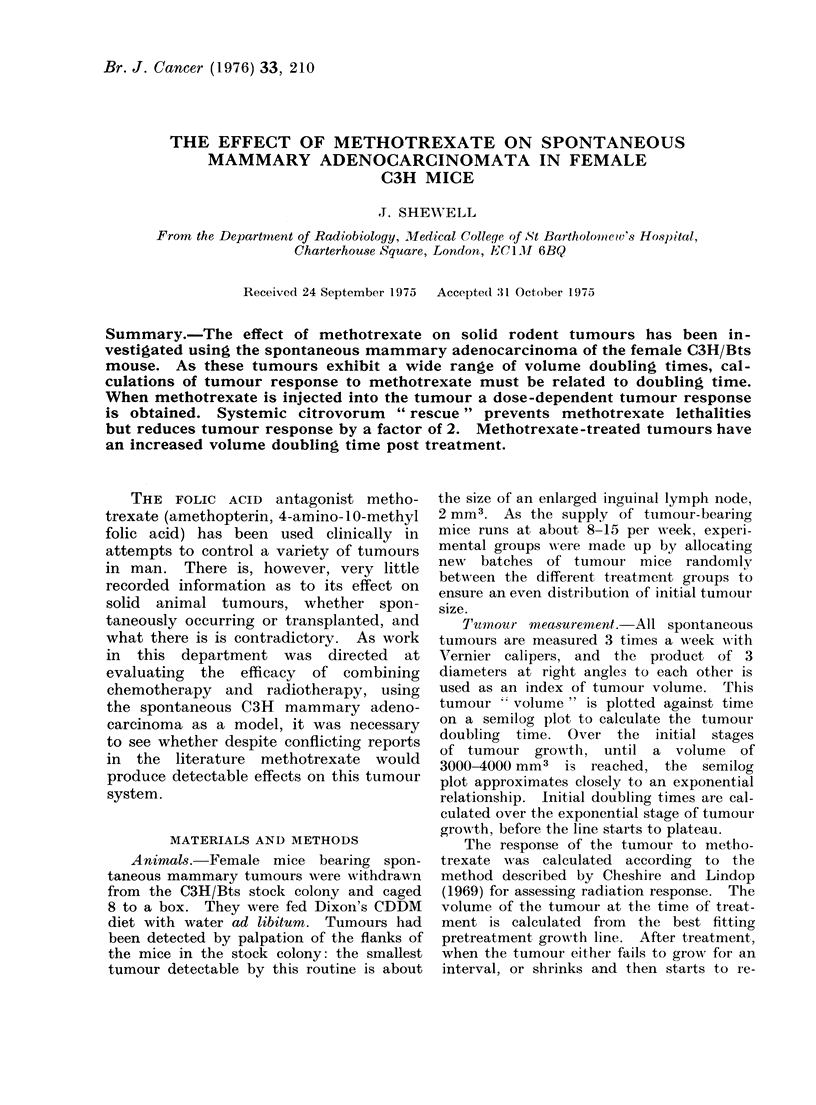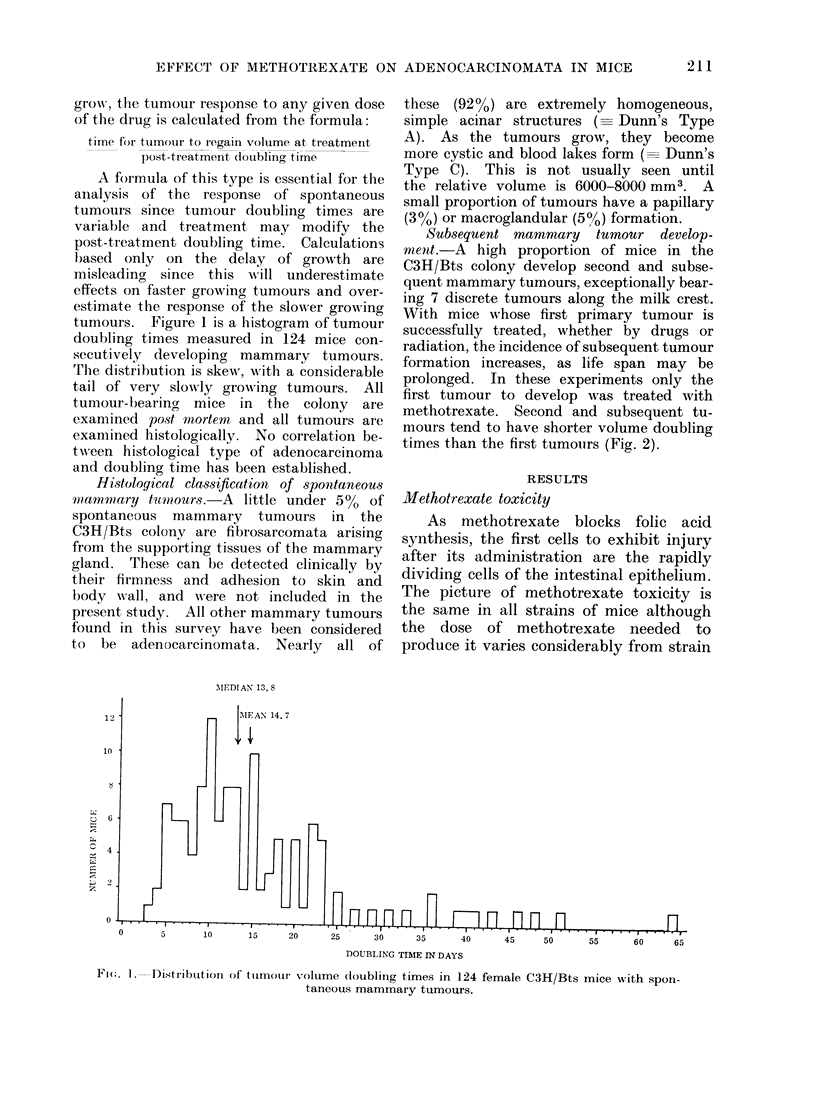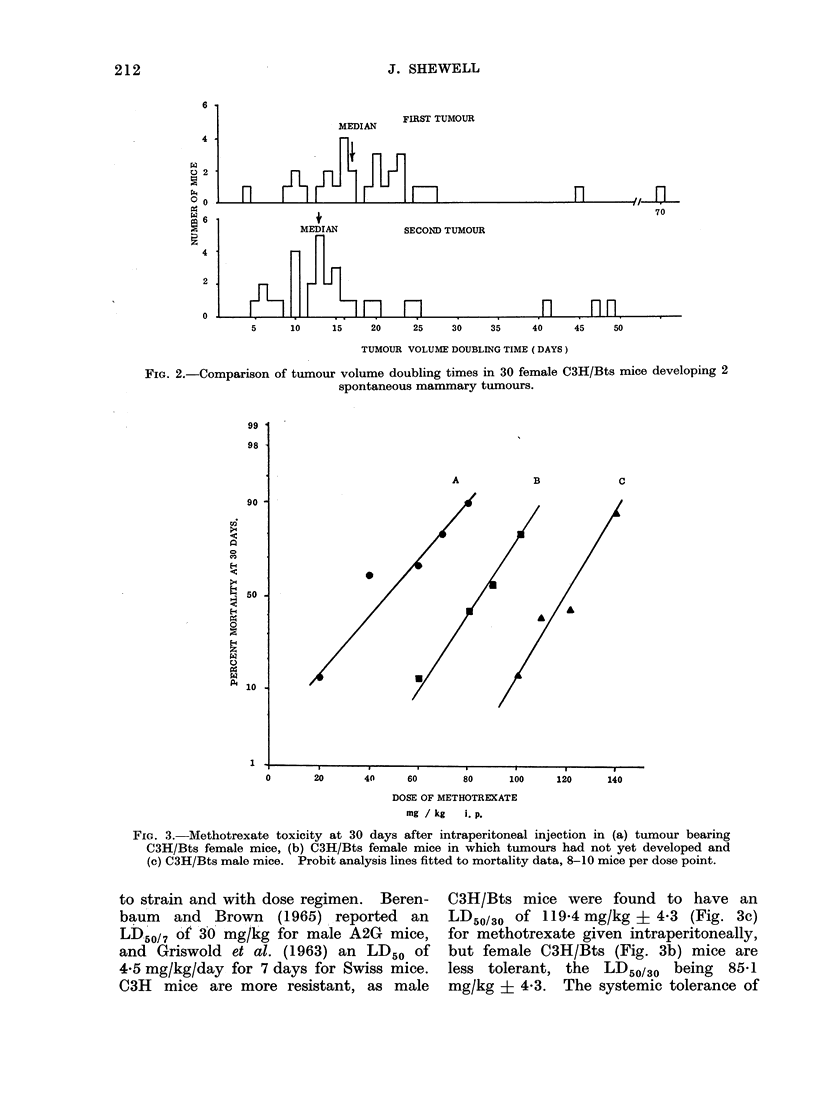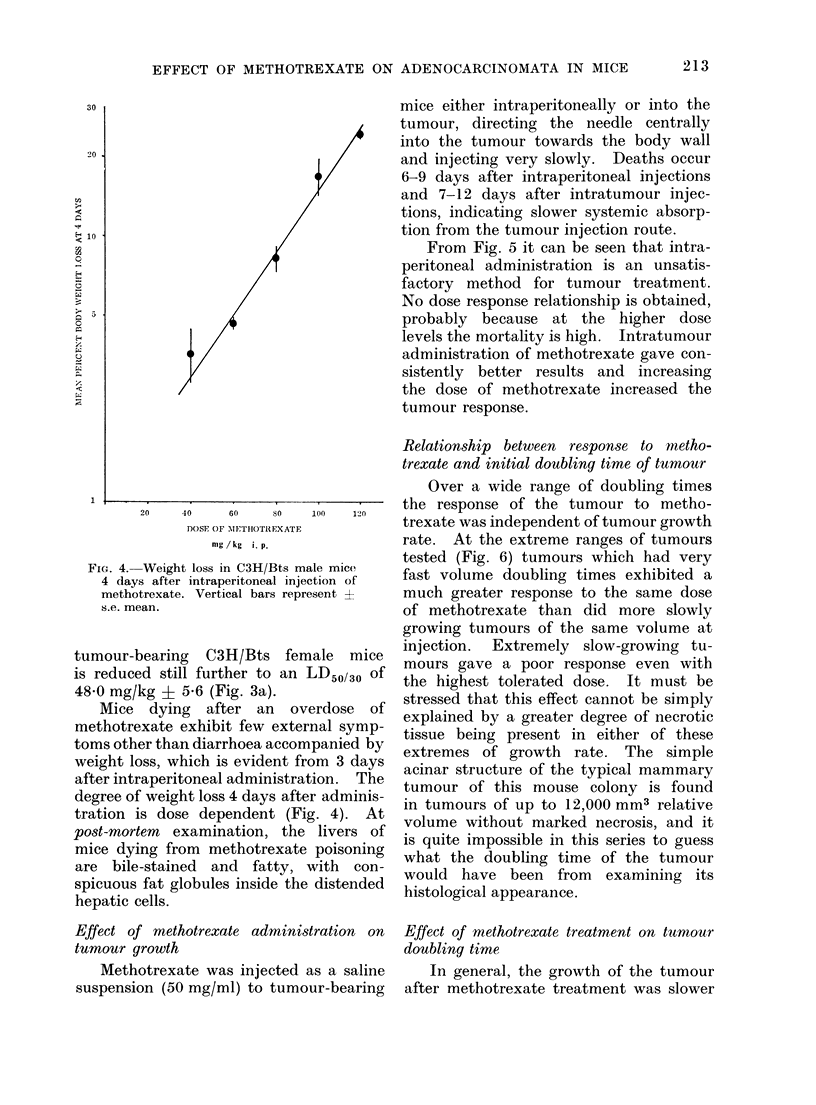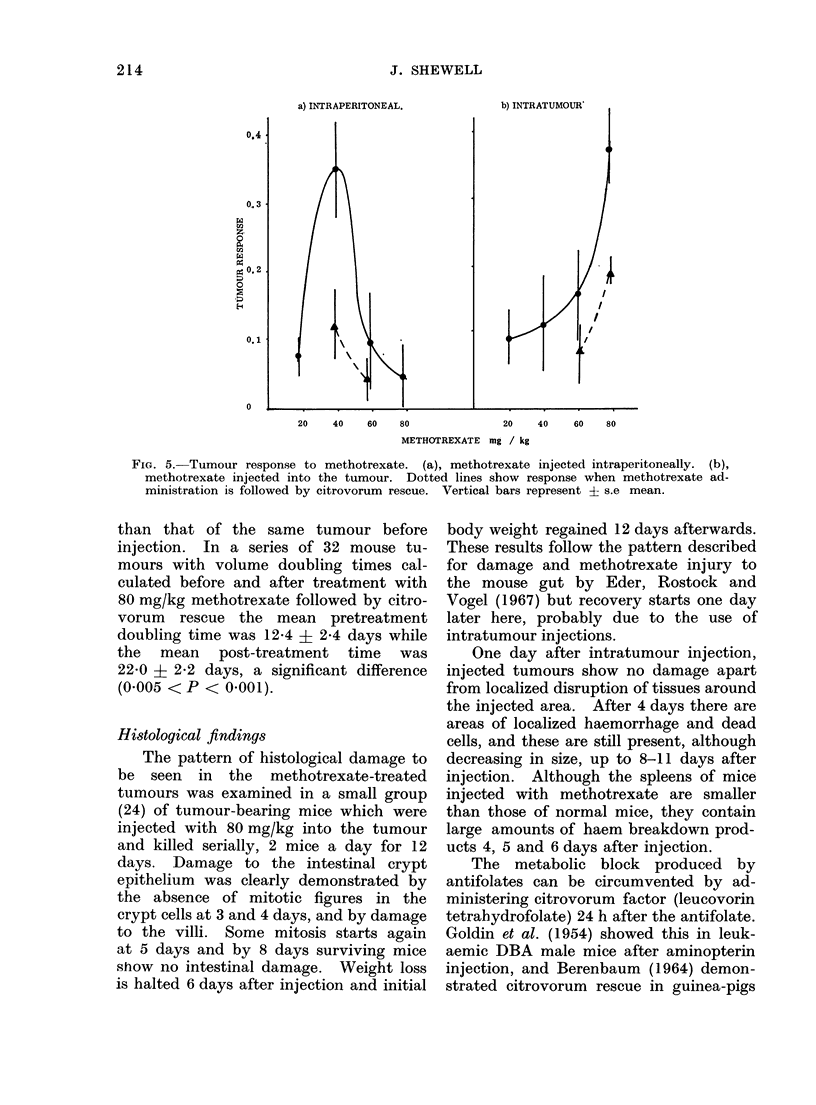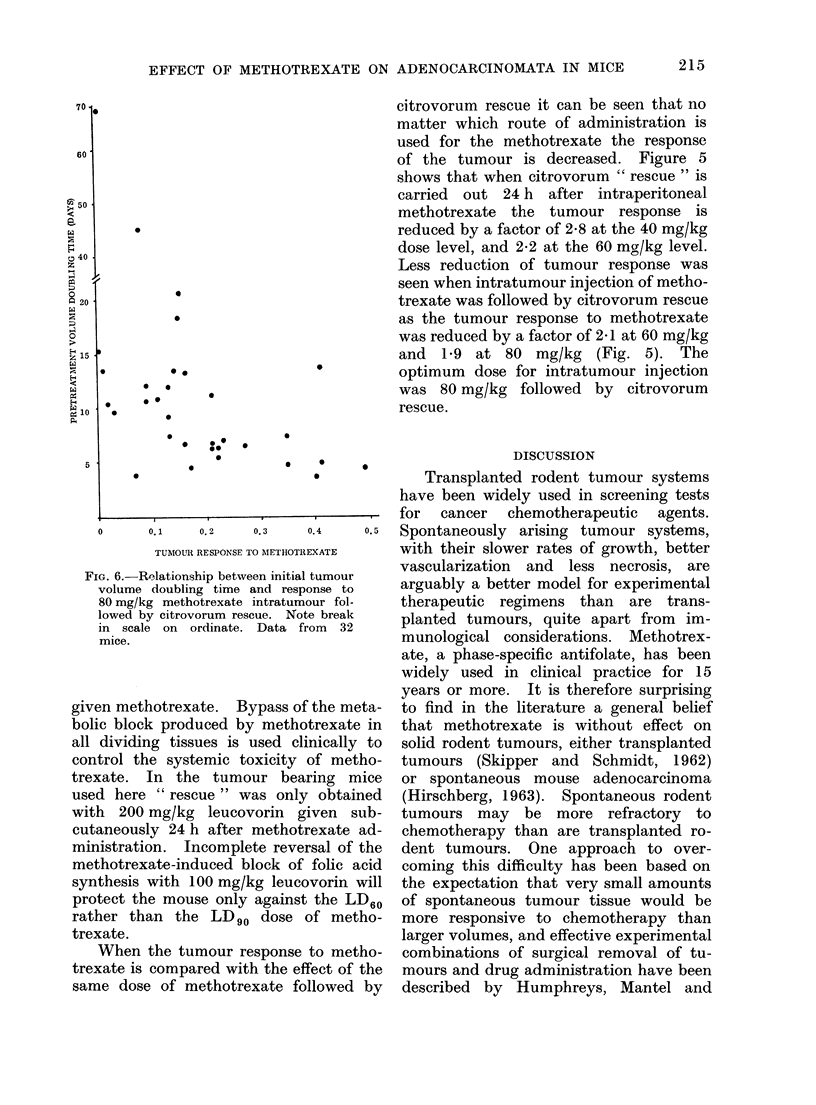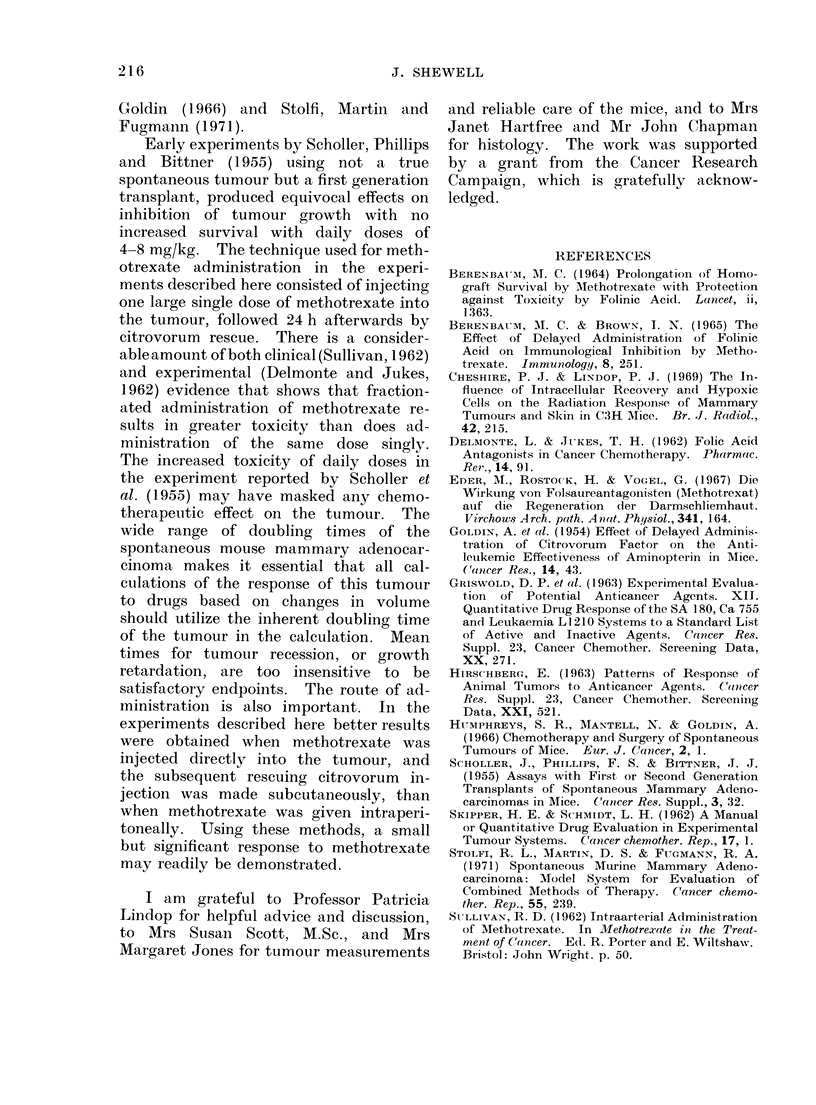# The effect of methotrexate on spontaneous mammary adenocarcinomata in female C3H mice.

**DOI:** 10.1038/bjc.1976.27

**Published:** 1976-02

**Authors:** J. Shewell

## Abstract

The effect of methotrexate on solid rodent tumours has been investigated using the spontaneous mammary adenocarcinoma of the female C3H/Bts mouse. As these tumours exhibit a wide range of volume doubling times, calculations of tumour response to methotrexate must be related to doubling time. When methotrexate is injected into the tumour a dose-dependent tumour response is obtained. Systemic citrovorum "rescue" prevents methotrexate lethalities but reduces tumour response by a factor of 2. Methotrexate-treated tumours have an increased volume doubling time post treatment.


*Br. J. Cancer* (1976) **33**, 210

### THE EFFECT OF METHOTREXATE ON SPONTANEOUS MAMMARY ADENOCARCINOMATA IN FEMALE C3H MICE

J. SHEWELL

*From the Department of Radiobiology, Medical College of St Bartholomew's Hospital, Charterhouse Square, London, EC1M 6BQ*

Received 24 September 1975 Accepted 31 October 1975

**Summary.—The effect of methotrexate on solid rodent tumours has been investigated using the spontaneous mammary adenocarcinoma of the female C3H/Bts mouse. As these tumours exhibit a wide range of volume doubling times, calculations of tumour response to methotrexate must be related to doubling time. When methotrexate is injected into the tumour a dose-dependent tumour response is obtained. Systemic citrovorum "rescue" prevents methotrexate lethalities but reduces tumour response by a factor of 2. Methotrexate-treated tumours have an increased volume doubling time post treatment.**

THE FOLIC ACID antagonist methotrexate (amethopterin, 4-amino-10-methyl folic acid) has been used clinically in attempts to control a variety of tumours in man. There is, however, very little recorded information as to its effect on solid animal tumours, whether spontaneously occurring or transplanted, and what there is is contradictory. As work in this department was directed at evaluating the efficacy of combining chemotherapy and radiotherapy, using the spontaneous C3H mammary adenocarcinoma as a model, it was necessary to see whether despite conflicting reports in the literature methotrexate would produce detectable effects on this tumour system.

### MATERIALS AND METHODS

*Animals.*—Female mice bearing spontaneous mammary tumours were withdrawn from the C3H/Bts stock colony and caged 8 to a box. They were fed Dixon's CDDM diet with water *ad libitum*. Tumours had been detected by palpation of the flanks of the mice in the stock colony: the smallest tumour detectable by this routine is about the size of an enlarged inguinal lymph node, 2 mm$^3$. As the supply of tumour-bearing mice runs at about 8–15 per week, experimental groups were made up by allocating new batches of tumour mice randomly between the different treatment groups to ensure an even distribution of initial tumour size.

*Tumour measurement.*—All spontaneous tumours are measured 3 times a week with Vernier calipers, and the product of 3 diameters at right angles to each other is used as an index of tumour volume. This tumour "volume" is plotted against time on a semilog plot to calculate the tumour doubling time. Over the initial stages of tumour growth, until a volume of 3000–4000 mm$^3$ is reached, the semilog plot approximates closely to an exponential relationship. Initial doubling times are calculated over the exponential stage of tumour growth, before the line starts to plateau.

The response of the tumour to methotrexate was calculated according to the method described by Cheshire and Lindop (1969) for assessing radiation response. The volume of the tumour at the time of treatment is calculated from the best fitting pretreatment growth line. After treatment, when the tumour either fails to grow for an interval, or shrinks and then starts to regrow, the tumour response to any given dose of the drug is calculated from the formula:

$$\frac{\text{time for tumour to regain volume at treatment}}{\text{post-treatment doubling time}}$$

A formula of this type is essential for the analysis of the response of spontaneous tumours since tumour doubling times are variable and treatment may modify the post-treatment doubling time. Calculations based only on the delay of growth are misleading since this will underestimate effects on faster growing tumours and overestimate the response of the slower growing tumours. Figure 1 is a histogram of tumour doubling times measured in 124 mice consecutively developing mammary tumours. The distribution is skew, with a considerable tail of very slowly growing tumours. All tumour-bearing mice in the colony are examined *post mortem* and all tumours are examined histologically. No correlation between histological type of adenocarcinoma and doubling time has been established.

*Histological classification of spontaneous mammary tumours.*—A little under 5% of spontaneous mammary tumours in the C3H/Bts colony are fibrosarcomata arising from the supporting tissues of the mammary gland. These can be detected clinically by their firmness and adhesion to skin and body wall, and were not included in the present study. All other mammary tumours found in this survey have been considered to be adenocarcinomata. Nearly all of these (92%) are extremely homogeneous, simple acinar structures (= Dunn's Type A). As the tumours grow, they become more cystic and blood lakes form (= Dunn's Type C). This is not usually seen until the relative volume is 6000–8000 mm$^3$. A small proportion of tumours have a papillary (3%) or macroglandular (5%) formation.

*Subsequent mammary tumour development.*—A high proportion of mice in the C3H/Bts colony develop second and subsequent mammary tumours, exceptionally bearing 7 discrete tumours along the milk crest. With mice whose first primary tumour is successfully treated, whether by drugs or radiation, the incidence of subsequent tumour formation increases, as life span may be prolonged. In these experiments only the first tumour to develop was treated with methotrexate. Second and subsequent tumours tend to have shorter volume doubling times than the first tumours (Fig. 2).

### RESULTS

### *Methotrexate toxicity*

As methotrexate blocks folic acid synthesis, the first cells to exhibit injury after its administration are the rapidly dividing cells of the intestinal epithelium. The picture of methotrexate toxicity is the same in all strains of mice although the dose of methotrexate needed to produce it varies considerably from strain

FIG. 1.—Distribution of tumour volume doubling times in 124 female C3H/Bts mice with spontaneous mammary tumours.

Fig. 2.—Comparison of tumour volume doubling times in 30 female C3H/Bts mice developing 2 spontaneous mammary tumours.

Fig. 3.—Methotrexate toxicity at 30 days after intraperitoneal injection in (a) tumour bearing C3H/Bts female mice, (b) C3H/Bts female mice in which tumours had not yet developed and (c) C3H/Bts male mice. Probit analysis lines fitted to mortality data, 8–10 mice per dose point.

to strain and with dose regimen. Berenbaum and Brown (1965) reported an $LD_{50/7}$ of 30 mg/kg for male A2G mice, and Griswold *et al.* (1963) an $LD_{50}$ of 4·5 mg/kg/day for 7 days for Swiss mice. C3H mice are more resistant, as male C3H/Bts mice were found to have an $LD_{50/30}$ of 119·4 mg/kg $\pm$ 4·3 (Fig. 3c) for methotrexate given intraperitoneally, but female C3H/Bts (Fig. 3b) mice are less tolerant, the $LD_{50/30}$ being 85·1 mg/kg $\pm$ 4·3. The systemic tolerance of

FIG. 4.—Weight loss in C3H/Bts male mice 4 days after intraperitoneal injection of methotrexate. Vertical bars represent ± s.e. mean.

tumour-bearing C3H/Bts female mice is reduced still further to an $LD_{50/30}$ of 48·0 mg/kg ± 5·6 (Fig. 3a).

Mice dying after an overdose of methotrexate exhibit few external symptoms other than diarrhoea accompanied by weight loss, which is evident from 3 days after intraperitoneal administration. The degree of weight loss 4 days after administration is dose dependent (Fig. 4). At *post-mortem* examination, the livers of mice dying from methotrexate poisoning are bile-stained and fatty, with conspicuous fat globules inside the distended hepatic cells.

*Effect of methotrexate administration on tumour growth*

Methotrexate was injected as a saline suspension (50 mg/ml) to tumour-bearing mice either intraperitoneally or into the tumour, directing the needle centrally into the tumour towards the body wall and injecting very slowly. Deaths occur 6–9 days after intraperitoneal injections and 7–12 days after intratumour injections, indicating slower systemic absorption from the tumour injection route.

From Fig. 5 it can be seen that intraperitoneal administration is an unsatisfactory method for tumour treatment. No dose response relationship is obtained, probably because at the higher dose levels the mortality is high. Intratumour administration of methotrexate gave consistently better results and increasing the dose of methotrexate increased the tumour response.

*Relationship between response to methotrexate and initial doubling time of tumour*

Over a wide range of doubling times the response of the tumour to methotrexate was independent of tumour growth rate. At the extreme ranges of tumours tested (Fig. 6) tumours which had very fast volume doubling times exhibited a much greater response to the same dose of methotrexate than did more slowly growing tumours of the same volume at injection. Extremely slow-growing tumours gave a poor response even with the highest tolerated dose. It must be stressed that this effect cannot be simply explained by a greater degree of necrotic tissue being present in either of these extremes of growth rate. The simple acinar structure of the typical mammary tumour of this mouse colony is found in tumours of up to 12,000 $mm^3$ relative volume without marked necrosis, and it is quite impossible in this series to guess what the doubling time of the tumour would have been from examining its histological appearance.

*Effect of methotrexate treatment on tumour doubling time*

In general, the growth of the tumour after methotrexate treatment was slower

FIG. 5.—Tumour response to methotrexate. (a), methotrexate injected intraperitoneally. (b), methotrexate injected into the tumour. Dotted lines show response when methotrexate administration is followed by citrovorum rescue. Vertical bars represent $\pm$ s.e mean.

than that of the same tumour before injection. In a series of 32 mouse tumours with volume doubling times calculated before and after treatment with 80 mg/kg methotrexate followed by citrovorum rescue the mean pretreatment doubling time was $12{\cdot}4 \pm 2{\cdot}4$ days while the mean post-treatment time was $22{\cdot}0 \pm 2{\cdot}2$ days, a significant difference ($0{\cdot}005 < P < 0{\cdot}001$).

### *Histological findings*

The pattern of histological damage to be seen in the methotrexate-treated tumours was examined in a small group (24) of tumour-bearing mice which were injected with 80 mg/kg into the tumour and killed serially, 2 mice a day for 12 days. Damage to the intestinal crypt epithelium was clearly demonstrated by the absence of mitotic figures in the crypt cells at 3 and 4 days, and by damage to the villi. Some mitosis starts again at 5 days and by 8 days surviving mice show no intestinal damage. Weight loss is halted 6 days after injection and initial body weight regained 12 days afterwards. These results follow the pattern described for damage and methotrexate injury to the mouse gut by Eder, Rostock and Vogel (1967) but recovery starts one day later here, probably due to the use of intratumour injections.

One day after intratumour injection, injected tumours show no damage apart from localized disruption of tissues around the injected area. After 4 days there are areas of localized haemorrhage and dead cells, and these are still present, although decreasing in size, up to 8–11 days after injection. Although the spleens of mice injected with methotrexate are smaller than those of normal mice, they contain large amounts of haem breakdown products 4, 5 and 6 days after injection.

The metabolic block produced by antifolates can be circumvented by administering citrovorum factor (leucovorin tetrahydrofolate) 24 h after the antifolate. Goldin *et al.* (1954) showed this in leukaemic DBA male mice after aminopterin injection, and Berenbaum (1964) demonstrated citrovorum rescue in guinea-pigs

FIG. 6.—Relationship between initial tumour volume doubling time and response to 80 mg/kg methotrexate intratumour followed by citrovorum rescue. Note break in scale on ordinate. Data from 32 mice.

given methotrexate. Bypass of the metabolic block produced by methotrexate in all dividing tissues is used clinically to control the systemic toxicity of methotrexate. In the tumour bearing mice used here " rescue " was only obtained with 200 mg/kg leucovorin given subcutaneously 24 h after methotrexate administration. Incomplete reversal of the methotrexate-induced block of folic acid synthesis with 100 mg/kg leucovorin will protect the mouse only against the $LD_{60}$ rather than the $LD_{90}$ dose of methotrexate.

When the tumour response to methotrexate is compared with the effect of the same dose of methotrexate followed by citrovorum rescue it can be seen that no matter which route of administration is used for the methotrexate the response of the tumour is decreased. Figure 5 shows that when citrovorum " rescue " is carried out 24 h after intraperitoneal methotrexate the tumour response is reduced by a factor of 2·8 at the 40 mg/kg dose level, and 2·2 at the 60 mg/kg level. Less reduction of tumour response was seen when intratumour injection of methotrexate was followed by citrovorum rescue as the tumour response to methotrexate was reduced by a factor of 2·1 at 60 mg/kg and 1·9 at 80 mg/kg (Fig. 5). The optimum dose for intratumour injection was 80 mg/kg followed by citrovorum rescue.

### DISCUSSION

Transplanted rodent tumour systems have been widely used in screening tests for cancer chemotherapeutic agents. Spontaneously arising tumour systems, with their slower rates of growth, better vascularization and less necrosis, are arguably a better model for experimental therapeutic regimens than are transplanted tumours, quite apart from immunological considerations. Methotrexate, a phase-specific antifolate, has been widely used in clinical practice for 15 years or more. It is therefore surprising to find in the literature a general belief that methotrexate is without effect on solid rodent tumours, either transplanted tumours (Skipper and Schmidt, 1962) or spontaneous mouse adenocarcinoma (Hirschberg, 1963). Spontaneous rodent tumours may be more refractory to chemotherapy than are transplanted rodent tumours. One approach to overcoming this difficulty has been based on the expectation that very small amounts of spontaneous tumour tissue would be more responsive to chemotherapy than larger volumes, and effective experimental combinations of surgical removal of tumours and drug administration have been described by Humphreys, Mantel and

Goldin (1966) and Stolfi, Martin and Fugmann (1971).

Early experiments by Scholler, Phillips and Bittner (1955) using not a true spontaneous tumour but a first generation transplant, produced equivocal effects on inhibition of tumour growth with no increased survival with daily doses of 4–8 mg/kg. The technique used for methotrexate administration in the experiments described here consisted of injecting one large single dose of methotrexate into the tumour, followed 24 h afterwards by citrovorum rescue. There is a considerable amount of both clinical (Sullivan, 1962) and experimental (Delmonte and Jukes, 1962) evidence that shows that fractionated administration of methotrexate results in greater toxicity than does administration of the same dose singly. The increased toxicity of daily doses in the experiment reported by Scholler *et al.* (1955) may have masked any chemotherapeutic effect on the tumour. The wide range of doubling times of the spontaneous mouse mammary adenocarcinoma makes it essential that all calculations of the response of this tumour to drugs based on changes in volume should utilize the inherent doubling time of the tumour in the calculation. Mean times for tumour recession, or growth retardation, are too insensitive to be satisfactory endpoints. The route of administration is also important. In the experiments described here better results were obtained when methotrexate was injected directly into the tumour, and the subsequent rescuing citrovorum injection was made subcutaneously, than when methotrexate was given intraperitoneally. Using these methods, a small but significant response to methotrexate may readily be demonstrated.

I am grateful to Professor Patricia Lindop for helpful advice and discussion, to Mrs Susan Scott, M.Sc., and Mrs Margaret Jones for tumour measurements and reliable care of the mice, and to Mrs Janet Hartfree and Mr John Chapman for histology. The work was supported by a grant from the Cancer Research Campaign, which is gratefully acknowledged.

### REFERENCES

BERENBAUM, M. C. (1964) Prolongation of Homograft Survival by Methotrexate with Protection against Toxicity by Folinic Acid. *Lancet*, ii, 1363.

BERENBAUM, M. C. & BROWN, I. N. (1965) The Effect of Delayed Administration of Folinic Acid on Immunological Inhibition by Methotrexate. *Immunology*, **8**, 251.

CHESHIRE, P. J. & LINDOP, P. J. (1969) The Influence of Intracellular Recovery and Hypoxic Cells on the Radiation Response of Mammary Tumours and Skin in C3H Mice. *Br. J. Radiol.*, **42**, 215.

DELMONTE, L. & JUKES, T. H. (1962) Folic Acid Antagonists in Cancer Chemotherapy. *Pharmac. Rev.*, **14**, 91.

EDER, M., ROSTOCK, H. & VOGEL, G. (1967) Die Wirkung von Folsaureantagonisten (Methotrexat) auf die Regeneration der Darmschliemhaut. *Virchows Arch. path. Anat. Physiol.*, **341**, 164.

GOLDIN, A. *et al.* (1954) Effect of Delayed Administration of Citrovorum Factor on the Antileukemic Effectiveness of Aminopterin in Mice. *Cancer Res.*, **14**, 43.

GRISWOLD, D. P. *et al.* (1963) Experimental Evaluation of Potential Anticancer Agents. XII. Quantitative Drug Response of the SA 180, Ca 755 and Leukaemia L1210 Systems to a Standard List of Active and Inactive Agents. *Cancer Res.* Suppl. 23, Cancer Chemother. Screening Data, **XX**, 271.

HIRSCHBERG, E. (1963) Patterns of Response of Animal Tumors to Anticancer Agents. *Cancer Res.* Suppl. 23, Cancer Chemother. Screening Data, **XXI**, 521.

HUMPHREYS, S. R., MANTELL, N. & GOLDIN, A. (1966) Chemotherapy and Surgery of Spontaneous Tumours of Mice. *Eur. J. Cancer*, **2**, 1.

SCHOLLER, J., PHILLIPS, F. S. & BITTNER, J. J. (1955) Assays with First or Second Generation Transplants of Spontaneous Mammary Adenocarcinomas in Mice. *Cancer Res.* Suppl., **3**, 32.

SKIPPER, H. E. & SCHMIDT, L. H. (1962) A Manual or Quantitative Drug Evaluation in Experimental Tumour Systems. *Cancer chemother. Rep.*, **17**, 1.

STOLFI, R. L., MARTIN, D. S. & FUGMANN, R. A. (1971) Spontaneous Murine Mammary Adenocarcinoma: Model System for Evaluation of Combined Methods of Therapy. *Cancer chemother. Rep.*, **55**, 239.

SULLIVAN, R. D. (1962) Intraarterial Administration of Methotrexate. In *Methotrexate in the Treatment of Cancer.* Ed. R. Porter and E. Wiltshaw. Bristol: John Wright. p. 50.